# Decreased expression of microRNA-320a promotes proliferation and invasion of non-small cell lung cancer cells by increasing VDAC1 expression

**DOI:** 10.18632/oncotarget.9943

**Published:** 2016-06-11

**Authors:** Guanxin Zhang, Gengxi Jiang, Chong Wang, Keng Zhong, Jiajun Zhang, Qing Xue, Xin Li, Hai Jin, Bailing Li

**Affiliations:** ^1^ Department of Cardiothoracic Surgery, Changhai Hospital, Second Military Medical University, Shanghai 200433, P.R. China

**Keywords:** non-small cell lung cancer, VDAC1, mRNA-320a, cell proliferation, invasion

## Abstract

Accumulating evidence indicates that Voltage Dependent Anion Channel 1 (VDAC1) correlates with the initiation and progression of non-small cell lung cancer (NSCLC). However, the regulatory mechanism of VDAC1 in NSCLC remains unclear. Previous studies have reported that expression of miR-320a was decreased in human primary squamous cell lung carcinoma, which prompted us to investigate whether there is a functional link between decreased miR-320a and a high expression of VDAC1. In the present report, using computational analysis, we first show that miR-320a has a potential binding site on VDAC1 mRNA, and expression of miR-320a was decreased in NSCLC cell lines. Using gain-of-function and rescue experiments, we demonstrate that VDAC1 is a direct target of miR-320a in NSCLC cells, and miR-320a inhibits VDAC1 expression in NSCLC cells. Further we show that MiR-320a was significantly decreased in NSCLC tissues compared with adjacent non-tumor tissues, and MiR-320a level is negatively correlated with VDAC1 in NSCLC tissues by Pearson's correlation coefficient analysis. Moreover, using cellular ATP assay, we found that suppression of VDAC1 expression may inhibit cell proliferation and invasion of NSCLC by decreasing cell energy and metabolism. Importantly, we showed that ectopic overexpression of miR-320a blocked tumor cell proliferation and invasion, both *in vitro* and *in vivo*, through inhibiting VDAC1. Our results suggest that reduced expression of miR-320a facilitates the development of NSCLCs by increasing VDAC1 expression. We identified a novel regulatory mechanism between miR-320a and VDAC1, and miR-320a may serve as a tumor suppressor gene and a promising therapeutic target of NSCLCs.

## INTRODUCTION

Lung cancer is one of the most common causes of cancer-associated deaths worldwide. Lung cancer is also the leading cancer in males, comprising 17% of the total new cancer cases and 23% of the total cancer deaths [1]. Non-small-cell lung cancer (NSCLC) accounts for 85-90% of lung cancers, while small-cell lung cancer (SCLC) has been decreasing in frequency over the last two decades [2]. NSCLC population has been grown quickly recently in China[3]. Although the strategies for prevention, diagnosis and treatment have been improved, 5-year survival after surgery is reported to be only 30-60% in NSCLC patients [4]. Therefore, elucidation of the mechanism underlying the initiation and progression of NSCLC is urgent and of great interest.

Voltage Dependent Anion Channel 1 (VDAC1) is a major component of the outer mitochondrial membrane (OMM), which plays an important role in the regulation of ATP/ADP exchange and respiratory control [5]. VDAC1 controls metabolic cross-talk between mitochondria and the rest of the cell by mediating the flux of ions, nucleotides, Ca(2+) and other metabolites across the OMM [6–8], indicating an essential role of VDAC1 in controlling cell energy and metabolic homeostasis and decreased expression of VDAC1 may result in suppression of cell energy and metabolism.

Along with regulating cellular energy production and metabolism, VDAC1 is involved in the process of mitochondria-mediated apoptosis by mediating the release of apoptotic proteins and interacting with anti-apoptotic proteins [9–11]. Oligomerization of VDAC1 in the inter-membranal space mediates the release of cytochrome c and AIF to the cytosol, subsequently leading to apoptotic cell death [9]. VDAC1 also regulates apoptosis as an anchor point for mitochondria-interacting proteins, such as hexokinase (HK), Bcl2 and Bcl-xL, some of which are highly expressed in many cancers [10–12, 13]. By binding to VDAC1, HK provides both a metabolic benefit and apoptosis-suppressive capacity that offers the cell a proliferative advantage and increases its resistance to chemotherapy [14]. Thus, these observations further implicate VDAC1 as an excellent target for impairing the re-programmed metabolism of cancer cells to evade apoptosis. However, the molecular mechanism underlying VDAC1 expression, especially in Non-small-cell lung cancer, remains largely unknown.

MicroRNAs (miRNAs) are a class of small non-coding RNAs (~22 nucleotides) that regulate gene expression by targeting promoters or mRNAs for transcriptional inhibition or translational repression [15]. MiRNAs play a role in regulating various biological processes, such as differentiation, proliferation, differentiation, and inhibiting apoptosis [16]. Accumulating evidences have suggested that miRNAs are involved in tumorigenesis and cancer progression, acting as either tumor suppressors or oncogenes, and have become potential biomarkers for cancer diagnosis, therapy, and prognosis [17]. Indeed, several deregulated miRNAs including miR-221, miR-222, miR-449a, miR-21, miR-205, miR-10b, miR-143 and miR-181a have been shown to regulate cell growth, apoptosis, migration and invasion [18–23], indicating an essential role of miRNAs in tumorigenesis of NSCLC.

In this current study, we show that miR-320 expression is markedly decreased in NSCLC, which in turn facilitates the development of NSCLC through increasing VDAC1 expression.

## RESULTS

### MiR-320 has a potential binding site on VDAC1 mRNA

**Figure 1:**
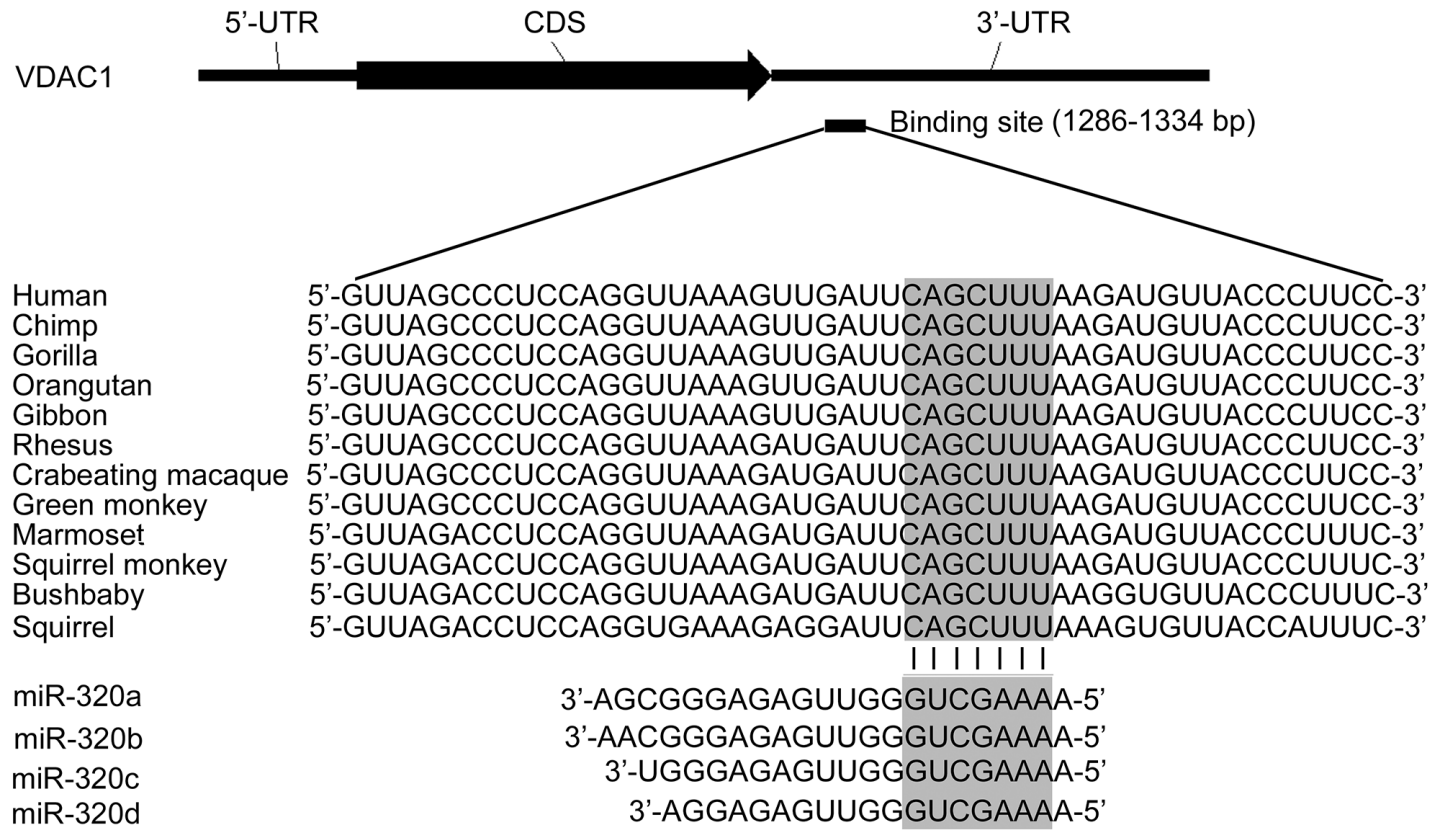
Bioinformatic analysis of miR-320 binding site in VDAC1 mRNA 3′-UTR Schematic diagram of VDAC1 mRNA 3′-UTR and the potential binding site for miR-320a. The upper panel shows one potential target site on 3′-UTR of VDAC1 and the lower panel shows multiple sequence alignment of miR-320 with the binding site on 3′UTR of VDAC1.

To predict the potential miRNAs targeting on VDAC1, the miRNA binding sites in the 3′-UTR of VDAC1 were computationally analyzed with PITA (genie.weizmann.ac.il/pubs/mir07/mir07_prediction.html), miRanda (www.microrna.org), Pictar (www.pictar.org) and TargetScan (www.targetscan.org). A conserved miR-320 (miR-320a, 320b, 320c and 320d) binding site exists in the 3′-UTR of VDAC1 across human (Homo sapiens (HSA)), chimpanzee (Pan troglodytes (PTR)), gorilla (Gorilla gorilla) and etc (Figure 1). Furthermore, the newly published CLASH data provided us with the direct evidence showing that both miR-320a and miR-320c have a potential binding site in 3′UTR of VDAC1 [24].

### MiR-320a is highly expressed in normal lung tissues and down-regulated in NSCLC cells

**Figure 2:**
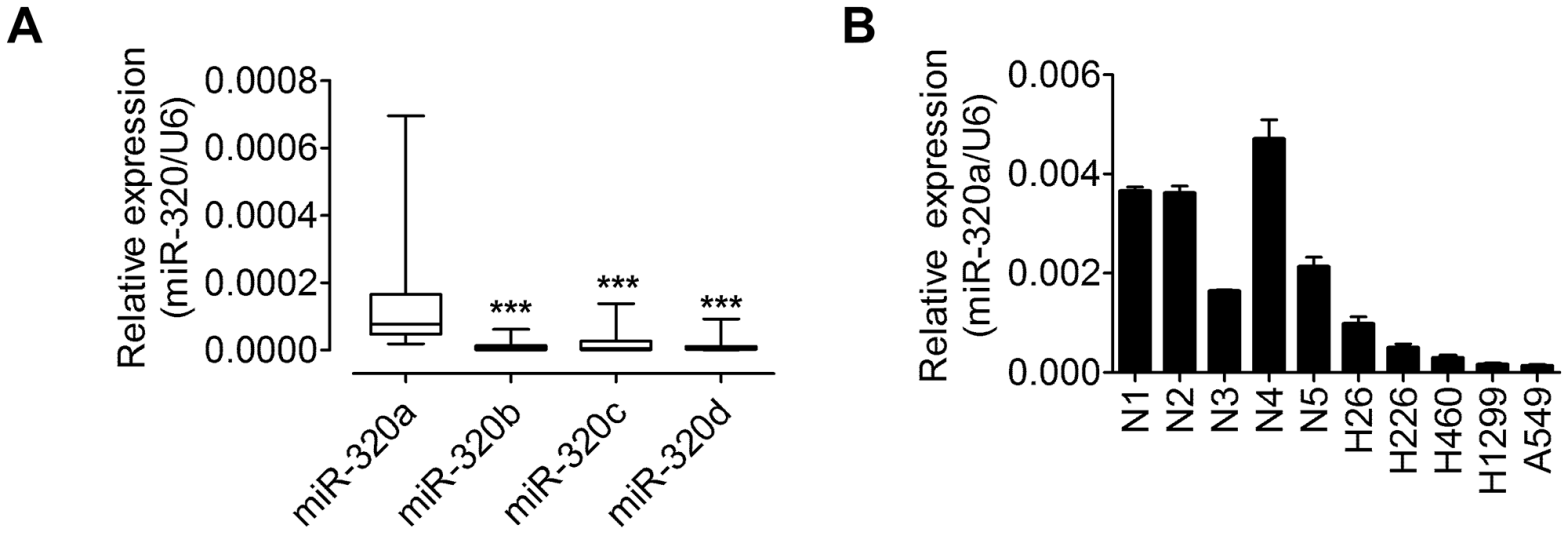
miR-320a is highly expressed in lung tissues and decreased in NSCLC cell lines **A.** qRT–PCR analysis of miR-320a, 320b, 320c and 320d expressions in 60 NSCLC corresponding non-tumor tissues. The expression of mRNA was normalized to U6. **B.** The expression levels of miR-320a were measured in 5 human NSCLC cell lines and 5 nromal lung tissues by qRT-PCR, and the expression levels of miR-320a were normalized to U6 RNA expression for subsequent analyses. ***p < 0.001.

To determine the levels of miR-320 family in lung tissues, total RNAs were extracted from 60 adjacent non-tumor tissues from NSCLC patients. The clinicopathologic features of 60 patients were listed in Table 1. The expression levels of miR-320a, 320b, 320c and 320d were analyzed using quantitative real-time polymerase chain reaction (qRT-PCR) and normalized against an endogenous control (U6 RNA). As shown in Figure 2A, miR-320a exhibits a higher expression level compared with miR-320b, 320c and 320d in lung tissues. Therefore, miR-320a was selected for further studies. Recently, miR-320 has been shown to be decreased in the squamous cell lung carcinoma tissues [25]. To determine whether miR-320a is down-regulated in NSCLC cells, expression levels of miR-320a were analyzed by qRT-PCR in five human NSCLC cell lines and five normal lung tissues. Our results demonstrated that miR-320a was down-regulated in five NSCLC cell lines, especially in A549 and H1299 cells (Figure 2B). Taken together, these findings indicate that miR320a may be critically involved in the development and progression of human NSCLC cells.

**Table 1:** Patients' characteristics of clinical-pathologic features

Characteristics	No. of patients (n=60)	Percent (%)
Age at diagnosis (year)		
≤ 65	40	66.7
> 65	20	33.3
Sex		
Male	35	58.3
Female	25	41.7
Tumor size (cm)		
≤ 3.0	45	75.0
> 3.0	15	25.0

### MiR-320a directly targets VDAC1 in NSCLC cells

**Figure 3:**
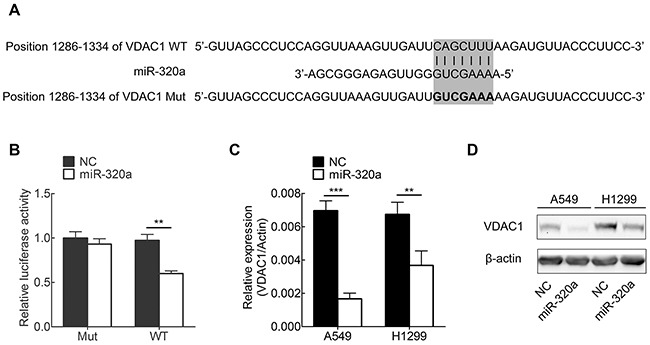
miR-320a targets VDAC1 in NSCLC cell lines **A.** Wild-type (WT) and mutant (Mut) of putative miR-320a targeting sequences in VDAC1 mRNA 3′-UTR. Mutant sequences were shown in bold type. **B.** Analysis of luciferase activity in HEK 293T cells. Cells were co-transfected with firefly luciferase reporter plasmid containing putative miR-320a targeting sequences. 48 hours after transfection, cell lysates were assayed for luciferase activity and normalized to Renilla luciferase activity. **C, D.** Effects of miR-320a on the endogenous VDAC1 expression levels. A549 and H1299 cells were co-transfected with miR-320a mimics and negative control oligonucleotides. 48 hours after transfection, mRNA and protein levels of VDAC1 were analyzed by qRT-PCR (C) and Western blotting (D). *p < 0.05, **p < 0.01, ***p < 0.001.

Based on our results showing that miR-320a was decreased in NSCLC cells, we attempted to determine whether miR-320a is capable of targeting and regulating VDAC1 expression in NSCLC cells. To this end, we created the luciferase reporter plasmids with wild type or mutant targeting sequence of VDAC1 mRNA (Figure 3A). The mimics of miR-320a were transfected into HEK 293T cells, and luciferase assay was used to assess the regulation of VDAC1 by miR-320a. Our results showed that overexpression of miR-320a significantly decreased the activity of luciferase fused with wild-type of VDAC1-3-UTR, but barely affected the activity of luciferase fused with mutated VDAC1-3′-UTR (Figure 3B). These results indicate that miR-320a may negatively regulate VDAC1 expression through targeting its 3′-UTR.

Furthermore, we examined whether the endogenous expression of VDAC1 in NSCLC cells is regulated by miR-320a. To this end, two NSCLC cell lines (A549 and H1299) were transfected with miR-320a mimics, and VDAC1 expression were determined by qRT-PCR and Western blotting. We found that VDAC1 expression was substantially decreased by mimics of miR-320a in NSCLC cells (Figure 3C, 3D).

### MiR-320a is negatively correlated with VDAC1 in NSCLC tissues

**Figure 4:**
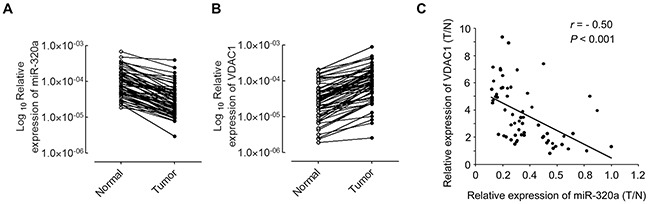
miR-320a negatively regulates VDAC1 mRNA expression in NSCLC samples **A.** qRT-PCR analysis of miR-320a expression in 60 pairs of NSCLC tissues and their corresponding non-tumor tissues. Expression of miR-320a was normalized to U6. **B.** qRT-PCR analysis of VDAC1 expression in NSCLC tissues and their corresponding non-tumor tissues as indicated in (A). The expression of VDAC1 was normalized to β-actin. **C.** A negative Spearman correlation between miR-320a and VDAC1 mRNA levels were found in 60 NSCLC samples. T, tumor tissues; N, adjacent non-tumor tissues. ***p < 0.001.

MiR-320a has been reported to be decreased in human primary squamous cell lung carcinoma [25], and over-expression of VDAC1 is associated with worse outcomes in a number of cancers [26]. Our results demonstrated that VDAC1 was negatively regulated by miR-320a in NSCLC cell lines. Thus, we investigated the correlation between miR-320a expression and mRNA levels of VDAC1 in NSCLC tissues. Total RNAs were extracted from 60 NSCLC tissues, and the expression levels of miR-320a and VDAC1 were analyzed by qRT-PCR. As shown in Figure 4A, miR-320a was significantly decreased in NSCLC tissues when compared with adjacent non-tumor tissues. Consistent with previous studies in other cancers [26], VDAC1 mRNA levels were significantly increased in NSCLC tissues versus adjacent non-tumor tissues (Figure 4B). After normalization to the expression value of normal tissues, RNA levels of miR-320a and mRNA levels of VDAC1 in NSCLC tissues were analyzed by Pearson's correlation coefficient analysis. We found that VDAC1 mRNA levels were negatively correlated with miR-320a expression levels in NSCLC tissues (r = −0.50, *p* < 0.001) (Figure 4C).

Collectively, our results indicate that miR-320a directly targets VDAC1 mRNA and negatively regulates expression of VDAC1 in both NSCLC cell lines and tissues.

### MiR-320a inhibits the proliferation and invasion of NSCLC cells *in vitro* by targeting VDAC1

Gene expression meta-analysis identified VDAC1 as a predictor of poor outcome in early stage NSCLC, and knockdown of VDAC1 expression has been shown to inhibit cancer cell proliferation and tumor growth [26, 27], which prompted us to hypothesize that miR-320a may affect NSCLC cell viability through VDAC1. To test this hypothesis, gain-of-function and rescue experiments were performed in NSCLC cells. We determined whether MiR-320a mimics inhibited the proliferation of NSCLC cells, and whether it could be rescued by transfecting VDAC1 cDNA if the inhibiting effect existed. In this regard, miR-320a mimics or VDAC1 cDNA was transiently transfected into A549 and H1299 cells, then the cell proliferation and matrigel invasion assays were performed.

**Figure 5:**
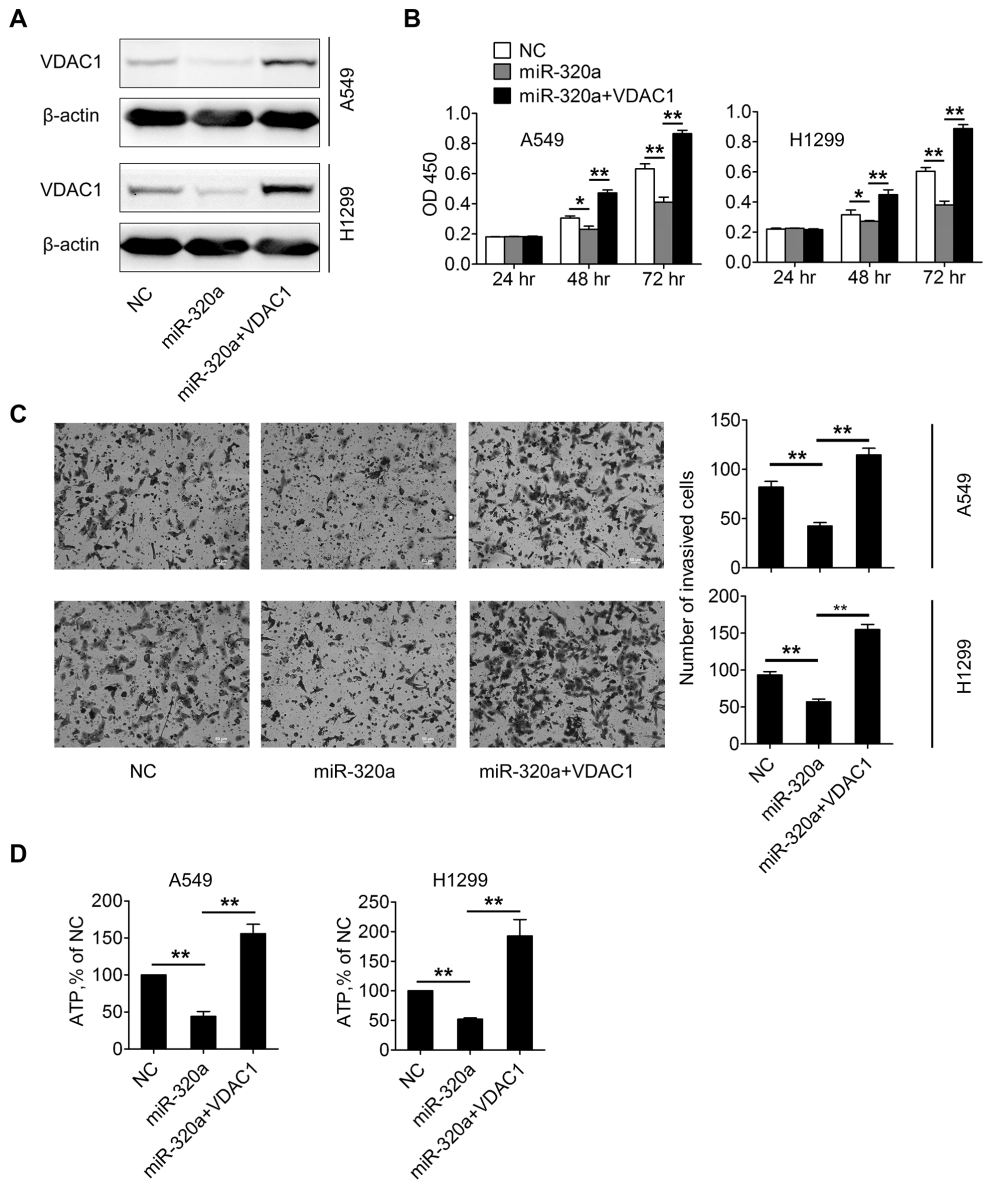
Up-regulation of miR-320a inhibits proliferation and invasion of NSCLC cells by targeting VDAC1 **A.** Western blotting analysis was performed to determine the expression level of VDAC1 after transfection of negative control (NC), miR-320a mimics (miR-320a) or miR-320a plus pGL3-VDAC1 (miR-320a + VDAC1). **B.** Cell proliferations were determined by CCK-8 assay at 24 h, 48 h, and 72 h after transfection of NC, miR-320a or miR-320a+VDAC1. **C.** The invasive ability of A549 and H1299 cells was evaluated by *in vitro* invasion assays after transfection of NC, miR-320a or miR-320a + VDAC1. **D.** ATP cellular levels were analyzed in A549 and H1299 cells after transfection of NC, miR-320a or miR-320a + VDAC1. (*p < 0.05, **p < 0.01).

We found that transfection of miR-320a in A549 and H1299 cells significantly suppressed the protein expression of VDAC1, while re-expression of VDAC1 by transfecting VDAC1 cDNA that cannot be targeted by miR-320a in miR-320a-tranfected cells rescued this suppression (Figure 5A), as determined by Western botting. Using cell proliferation assay, over-expression of miR-320a in A549 and H1299 cells resulted in significant suppression of cell proliferation, while re-expression of VDAC1 in miR-320a-tranfected cells significantly increased cell proliferation in A549 and H1299 cells (Figure 5B). In matrigel invasion assays, overexpression of miR-320a significantly decreased migration of A549 and H1299 cells, while re-expression of VDAC1 in miR-320a-tranfected cells significantly increased migration of A549 and H1299 cells (Figure 5C).

VDAC1 controls energy production and metabolic crosstalk between the cytosol and mitochondria [30]. To ascertain whether the decreased expression of hVDAC1 leading to inhibition of cell proliferation and invasion acts through a disruption of energy production, cellular ATP levels by mitochondria isolated from control, miR-320a-tranfected, and re-expression of VDAC1 in miR-320a-tranfected cells were compared. A549 and H1299 cells treated with miR-320a showed a decrease of cellular ATP levels as compared to controls, while re-expression of VDAC1 in miR-320a-tranfected cells significantly increased cellular ATP levels of A549 and H1299 cells (Figure 5D). These preliminary data prompted that down-regulation of VDAC1 expression may inhibit cell proliferation and invasion of NSCLC by decreasing cell energy and metabolism.

### MiR-320a suppresses tumor growth of NSCLC xenografts

**Figure 6:**
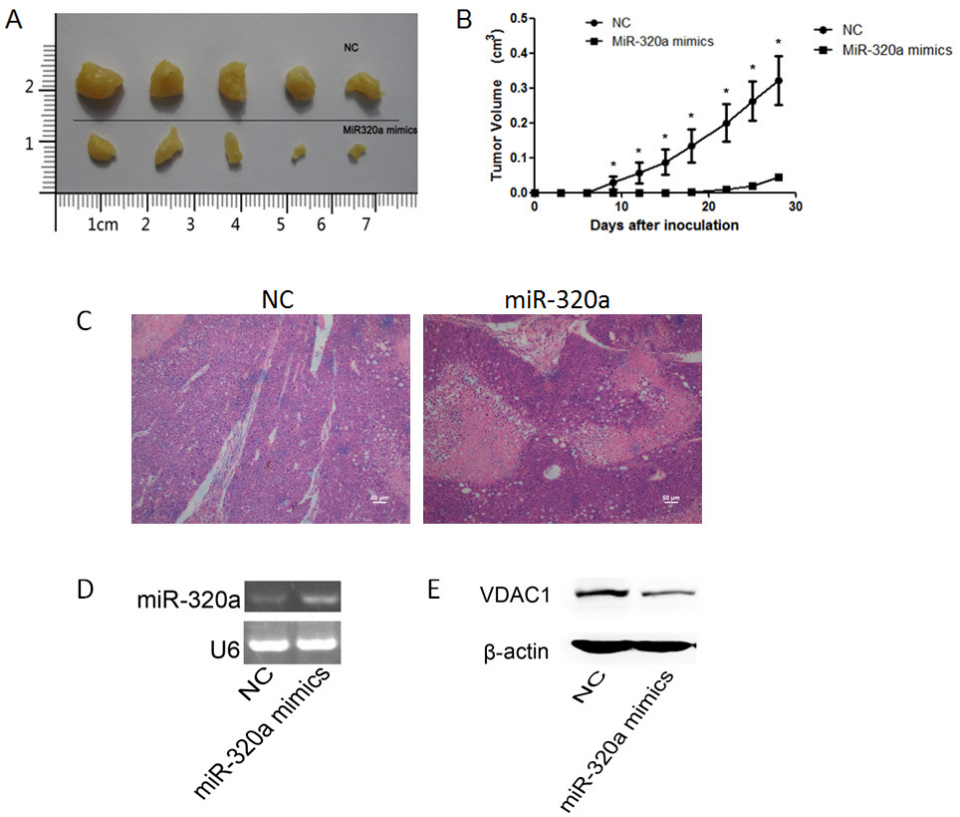
Effect of miR-320a over-expression on tumorigenicity **A.** Tumors from Nod/Scid mice 4 weeks after inoculation. **B.** The curve of tumor growth. Wilcoxon signed-ranks test was used for the comparison of tumor volumes. *p < 0.05 versus NC transfectants. **C.** H&E staining in xenograft tumors (Scale bars: 50 μm). **D.** The expression levels of miR-320a in miR-320a mimics transfected tumor cells group and NC group determined by RT-PCR. **E.** The protein level of VDAC1 was decreased in miRNA mimics transfected tumor cells group compared to NC group when overexpressing miR-320a.

An *in vivo* model was used to evaluate the effect of miR-320a overexpression on tumorigenicity. MiR-320a mimics and NC transfected A549 cells were injected subcutaneously into either side of the posterior flank of the same Nod/Scid mice. Five mice were used and tumor growth was examined every three days over a course of 4 weeks as described previously [28]. Our results showed that miR-320a mimics-transfected cells exhibited a significant reduction in the tumor size compared with NC transfectants, suggesting that increased miR-320a expression possesses a potential tumor suppressive effect (Figure 6A, 6B).

We also perform histologic staining to observe the pathological change in the xenograft tumors between miRNA mimics and NC transfected tumor cells groups. HE staining showed that there were more necrosis regions in miRNA mimics transfected tumor cells group than in NC group, indicating tumor cells' proliferation was suppressed when overexpressing miR-320a (Figure 6C).

Moreover, we examined the expression level of VDAC1 in the xenograft tumors using Western blotting experiments. We found the protein level of VDAC1 was decreased in miRNA mimics transfected tumor cells group compared to NC group when overexpressing miR-320a (Figure 6D, 6E).

Taken together, our results demonstrate that decreased expression of VDAC1 by miR-320a contributes to the suppression of the growth of NSCLC cells.

## DISCUSSION

Non-small-cell lung cancer (NSCLC) is the most common type of lung cancer. It is essential to elucidate the underlying mechanism that mediates the initiation and progression of NSCLC and identify potential therapeutic targets for treatment this disease. As a transporter of metabolites, VDAC1 contributes to the metabolic phenotype of cancer cells such as increased proliferation and invasiveness. Indeed, this protein is over-expressed in many cancer types, and silencing of VDAC1 expression induces an inhibition of tumor development. Gene expression meta-analysis also identifies VDAC1 as a predictor of poor outcome in early stage non-small cell lung cancer, and silencing VDAC1 expression by siRNA has been shown to inhibit cancer cell proliferation and tumor growth [26, 27, 29]. Conversely, high levels of VDAC1 expression may endow tumor cells with a selection advantage by facilitating energy dependent processes such as proliferation and invasiveness [30]. However, the molecular mechanism of how VDAC1 expression is regulated in NSCLC is largely unknown.

MiRNAs play a pivotal role in carcinogenesis either as oncogenes or as tumor suppressor genes, which mediate the cancer progression including proliferation, migration, invasion and apoptosis. Deregulation of miRNAs such as miR-221, miR-222, miR-449a, miR-21, miR-205, miR-10b, miR-143 and miR-181a in NSCLC has been shown to be a key factor in tumorigenesis [31]. Previous studies had reported that miR-320a was down-regulated in human primary squamous cell lung carcinoma [25]. Therefore, we want to know if there is a functional link between miR-320a and a higher expression of VDAC1 in NSCLC.

In the present report, using computational analysis we found that miR-320 family has a potential binding site on VDAC1 mRNA 3′-UTR. Based on the relative expression levels of miR-320 family in lung tissues, miR-320a was further selected to investigate its role in the development and progression of NSCLC. Our results confirmed that miR-320a is markedly decreased in five NSCLC cell lines, especially in A549 and H1299 cells. In order to determine whether miR-320a directly targets and regulates VDAC1 expression in NSCLC cells, we performed gain-of-function and rescue experiments. We found that transfection of miR-320a in A549 and H1299 cells significantly suppressed the protein expression of VDAC1, and this suppression could be rescued by transfecting VDAC1 cDNA in miR-320a-tranfected cells. Our results indicated that VDAC1 was a direct target of miR-320a in NSCLC cells, and miR-320a down-regulated VDAC1 expression in NSCLC cells. Further, we confirmed that over-expression of miR-320a in A549 and H1299 cells resulted in significant suppression of cell proliferation and decreased migration, and this suppression could be rescued after re-expression of VDAC1. Furthermore, we found MiR-320a was significantly decreased in NSCLC tissues versus adjacent non-tumor tissues, and its expression is negatively correlated with VDAC1 in NSCLC tissues by Pearson's correlation coefficient analysis. As far as we know, this is the first report of regulatory mechanism between miR-320a and VDAC1. By the way, because cell cycle progression was not changed in VDAC silencing cells according to previous report [32], we didn't perform cell cycle analysis in this study.

The role of VDAC1 as oncogene was found in many cancer lines or tissues [27, 32]. Wu et al reported that cervical cancer tissues with positive VDAC1 immunoreactivity exhibited deep stromal invasion and large tumor size, and cervical cancer patients with positive VDAC1 immunoreactivity displayed higher recurrence and poorer overall survival than those with negative VDAC1 [32]. Gene expression meta-analysis also identified VDAC1 as a predictor of poor outcome in the early stage of NSCLC [26]. VDAC1 also plays an important role in the regulation of ATP/ADP exchange and respiratory control, and VDAC1 controls energy production and metabolic crosstalk between the cytosol and mitochondria [5, 27, 30]. In order to show the downstream effects of VDAC suppression, we assume that decreased expression of VDAC1 leading to inhibition of cell proliferation and invasion acts through a disruption of energy production. In our study cellular ATP levels by mitochondria isolated from control, miR-320a-tranfected, and re-expression of VDAC1 in miR-320a-tranfected cells were compared. We found that A549 and H1299 cells treated with miR-320a showed a decrease of cellular ATP levels as compared to controls, while re-expression of VDAC1 in miR-320a-tranfected cells significantly increased cellular ATP levels of A549 and H1299 cells. Therefore, these preliminary data prompted that down-regulation of VDAC1 expression may inhibit cell proliferation and invasion of NSCLC by decreasing cell energy and metabolism. However, more evidences in the relation between VDAC1 and energy control, as well as cell proliferation and invasion in NSCLC, need be provided in the future.

Finally we used an *in vivo* model to evaluate the effect of miR-320a overexpression on tumorigenicity. Our results showed that miR-320a mimics-transfected cells exhibited a significant reduction in the tumor size compared with NC transfectants, suggesting that up-regulation of miR-320a expression possesses a potential tumor suppressive effect. HE staining showed that there were more necrosis regions in miRNA mimics transfected tumor cells group than in NC group, indicating tumor cells' proliferation was suppressed when overexpressing miR-320a. We also confirmed that the protein level of VDAC1 was decreased in miRNA mimics transfected tumor cells group compared to NC group when overexpressing miR-320a. These data further demonstrate that decreased VDAC1 expression by miR-320a contributes to the suppression of the growth of NSCLC cells.

In summary, our findings suggest that reduced expression of miR-320a facilitates the development of NSCLC by increasing VDAC1 expression. We identified a novel regulatory mechanism between miR-320a and VDAC1. MiR-320a may serve as a tumor suppressor gene in NSCLC pathogenesis, and miR-320a may be a promising therapeutic target in the treatment of NSCLC.

## MATERIALS AND METHODS

### Specimens and cell lines

60 non-small-cell lung cancer (NSCLC) tissues and adjacent non-tumor tissues were obtained in accordance with the protocol approved by the Ethics Committee of Shanghai Changhai Hospital (Shanghai, China). The patients received the necessary information concerning the study, and consent was obtained. Additionally, five normal lung tissues were obtained from adjacent lung tissues in traumatic lung injury patients. The human NSCLC cell lines A549, H26, H226, H460 and H1299 were purchased from American Type Culture Collection (ATCC) and were cultured in the supplemented media with ATCC recommendation. HEK-293T cells (ATCC) were cultured in DMEM media containing 10% FBS. Cells were cultured at 37°C in a humidified atmosphere containing 5% CO_2_.

### RNA isolation, RT-PCR and qRT-PCR

Total RNA was isolated from NSCLC tissues, adjacent non-tumor tissues, NSCLC cell lines, and xenograft tumors using Trizol according to the manufacturer's instructions. Purified mRNA and miRNAs were detected by qRT-PCR assay using All-in-One miRNA qRT-PCR Detection Kit (GeneCopoeia, USA). All primers were listed in Table 2. U6 small RNA was used as an internal control for normalization and quantification of miR-320 expression. β-actin was used as an internal control for normalization and quantification of VDAC1 expression.

**Table 2:** All primers used in this study

Name	Primer Sequence
U6 F	5′-CTCGCTTCGGCAGCACA-3′
U6 R	5′-AACGCTTCACGAATTTGCGT-3′
β-actin F	5′-CGTCTTCCCCTCCATCG-3′
β-actin R	5′-CTCGTTAATGTCACGCAC-3′
VDAC1 F	5′-ACGTATGCCGATCTTGGCAAA-3′
VDAC1 R	5′-TCAGGCCGTACTCAGTCCATC-3′
miR-320a F	5′-AAAAGCTGGGTTGAGAGGGCGA-3′
miR-320b F	5′-AAAAGCTGGGTTGAGAGGGCAA-3′
miR-320c F	5′-AAAAGCTGGGTTGAGAGGGT-3′
miR-320d F	5′-AAAAGCTGGGTTGAGAGGA-3′
VDAC1 (WT) F	5′-AAACTAGTTAGTGTATCTTTTAATGTTGTATGTCTGG-3′
VDAC1 (WT) R	5′-GGAAGCTTGGGTAACATCTTAAAGCTGAATCAAC-3′
VDAC1 (MUT) F	5′-AAACTAGTTAGTGTATCTTTTAATGTTGTATGTCTGG-3′
VDAC1 (MUT) R	5′-GGAAGCTTGGGTAACATCTTTTTCGACAATCAAC −3′
VDAC1 (pGL3) F	5′-AAAGGTACCATGGCTGTGCCACCCACGTATG-3′
VDAC1 (pGL3) R	5′-AAACTCGAGTTATGCTTGAAATTCCAGTCCTAG-3′

Abbreviations: F, forward primer; R, reverse primer; WT, wild type; MUT, mutant.

### Luciferase assays

Luciferase reporter plasmid was constructed by cloning human VDAC1 mRNA 3′-UTR into pMIR-Report construct (Ambion, Austin, USA). Wild type or mutant VDAC1 mRNA fragment was amplified and cloned into the luciferase reporter via Spe*I* and Hind*III* sites. All the primers were listed in Table 2. Luciferase reporter assays were performed using Dual-Luciferase Reporter Assay System (Promega). Briefly, HEK 293T cells plated in a 96-well plate were co-transfected with 50 nM miR-320a mimics or negative control oligonucleotides, 20 ng of firefly luciferase reporter and 10 ng of pRL-TK (Promega, USA) using the INTERFERin reagent (Polyplus-transfection, France). Cells were collected 24 hours after transfection for luciferase assay.

### Oligonucleotides and plasmids transfection

All miRNA sequences were got from miRBase (http://www.mirbase.org). RNA oligos were chemically synthesized and purified by Genepharma Co. Ltd., (Shanghai, China). Sequence of human miR-320a mimics was 5′-AAA AGC UGG GUU GAG AGG GCG A-3′. Negative control oligonucleotides for miRNA was 5′-CAG UAC UUU UGU GUA GUA CAA-3′. The transfections were performed with INTERFERin reagent (Polyplus-transfection). The final concentration of miRNA was 50 nM. To generate pGL3-VDAC1 constructs, the sequence of VDAC1 mRNA was amplified by the primers listed in Table 2. The fragments were inserted into pGL3 with the designed cutting sites: *Kpn*I and *Xho*I. The transfections were performed with INTERFERin reagent (Polyplus-transfection). The final concentration of plasmids was 100 ng.

### Cell proliferation assay

Cell proliferation was measured using the CCK-8 assay kit (Dojindo Corp., Japan) according to the manufacturer's protocol. For Cell Counting, harvested cells were seeded in 96-well plates at 1 × 10^4^ per well (n=4 for each time point) in a final volume of 100 μl. Cells were counted for 24, 48 and 72 hours after transfection. On the day of harvest, 10 μl CCK-8 was added to 90 μl of culture medium. The cells were subsequently incubated for 2 hr at 37°C and the optical density was measured at 450 nm. Three independent experiments were performed.

### In vitro invasion assays

The invasive ability of NSCLC cells was determined using 24-well transwell chambers coated with Matrigel (BD Pharmingen, San Jose, CA, USA). Chambers have upper and lower culture compartments that are separated by polycarbonate membranes with 8-Lm pores (Costar, Cambridge, MA, USA). Transfected cells in serum-free medium were seeded at 5×10^4^ in the top chamber and the bottom chamber was filled with RPMI-1640 containing 10% FBS as a chemoattractant. The chamber was incubated at 37°C in a humidified incubator containing 5% CO_2_. Twenty four hours later, cells that migrated to the underside of the membrane were fixed with 4% paraformaldehyde (Sigma Aldrich, St. Louis, MO), stained with crystal violet (Beyotime, Shanghai, China), imaged, and counted with a microscope (Leica, UK). All experiments were performed in triplicate.

### Determination of cellular ATP levels

Cellular ATP levels were estimated using the luciferase-based assay (CellTiter-Glo, Promega). 36 hours post-transfection, the cells were washed twice with PBS and seeded in 96-well plates at densities of 5 × 10^4^ cells/ml. ATP levels were assayed according to the manufacturer' s protocol as described previously [30].

### Gel electrophoresis and immunoblotting

Total cellular extracts (20 μg) were separated by a 4-20% Tris-glycine gel and then transferred to a PVDF membrane (Immobilon-P transfer membranes; Millipore Corp.) Following the transferring, the blots were blocked with 5% non-fat dry milk in PBS with 0.1% Tween-20 for 2 h and washed three times with PBS with 0.1% Tween-20 at 4°C. The blots were then probed with 1:200 dilution of primary antibody against VDAC1 (ab28777, Abcam, USA) and β-actin (c-11; Santa Cruz Biotechnology, USA). The blots were then probed with secondary antibodies for 1 h at 4°C, followed by washes in PBS with 0.1% Tween-20 and detected using an Odyssey Scanning system.

### Tumorigenicity assays

MiR-320a mimics and NC transfected A549 cells (1 × 10^6^) were suspended in 150 μl PBS and then injected subcutaneously into either side of the posterior flank of Nod/Scid mice (7 weeks old). Five Nod/Scid mice were injected and tumor growth was examined every three days for 4 weeks. Tumor volume (V) was monitored by measuring the length (L) and width (W) of the tumor with calipers and was calculated with the formula V = (L × W^2^) × 0.5 (28). Hematoxylin and eosin (H&E) staining were preformed according to the usual protocol. All mice received humane care according to the guidelines of Animal Care and Use Committees of Shanghai Changhai Hospital.

### Statistical analysis

SPSS 16.0 statistical software was used to perform statistical analyses. Data are presented as means ± standard deviation (s.d.). Data were subjected to Student's *t*-test and one-way ANOVA, and *p* < 0.05 was considered statistically significant.
